# Nationwide outbreak of Shiga toxin-producing *Escherichia coli* infections associated with frozen pizzas, France, 2022

**DOI:** 10.2807/1560-7917.ES.2026.31.8.2500506

**Published:** 2026-02-26

**Authors:** Catarina Krug, Nathalie Jourdan-Da Silva, Mathieu Tourdjman, Patricia Mariani-Kurkdjian, Aurélie Cointe, Sophie Lefèvre, Sophie Bélichon, Claire Postic, Marie Françon, Hubert Herber, Delphine Sergentet, Sarah Ganet, Alicia Faure-Bondat, Marion Debin, Charly Kengne-Kuetche, Henriette de Valk, Stéphane Bonacorsi, François-Xavier Weill, Gabrielle Jones, on behalf of the Outbreak Investigation Team

**Affiliations:** 1 Santé publique France, Infectious Diseases Division, the French Public Health Agency, Saint-Maurice, France; 2 ECDC Fellowship Programme, Field Epidemiology path (EPIET), European Centre for Disease Prevention and Control (ECDC), Stockholm, Sweden; 3 National associated Reference Centre for *Escherichia coli*, Department of Microbiology, Robert Debré Hospital, Assistance Publique – Hôpitaux de Paris (AP-HP), Paris, France; 4 Institut Pasteur, Université Paris Cité, National Reference Centre for *Escherichia coli*, *Shigella* and *Salmonella*, Paris, France; 5 General Directorate for Food (DGAL), Paris, France; 6 Directorate General for Competition Policy, Consumer Affairs and Fraud Control (DGCCRF), Paris, France; 7 French National Reference Laboratory for *Escherichia coli* Including Shiga Toxin-Producing *E. coli* (NRL-STEC), VetAgro Sup—Campus Vétérinaire, Université de Lyon, Marcy-l’Étoile, France; 8 Sorbonne Université, INSERM, Institut Pierre Louis d’Epidémiologie et de Santé Publique, IPLESP, F75012, Paris, France; 9 The members of the Outbreak Investigation Team are listed under Acknowledgements

**Keywords:** Children, Disease outbreaks, Food contamination, Flour, Shiga toxigenic *Escherichia coli*, Haemolytic-uraemic syndrome, frozen pizzas

## Abstract

In February 2022, we observed an increase in the number of paediatric patients with haemolytic uraemic syndrome (HUS) associated with Shiga toxin-producing *Escherichia coli* (STEC) in France. We interviewed cases or caretakers about food exposures, identified purchases on supermarket loyalty cards, conducted a case–control study, tested food samples and characterised isolates. We identified 59 cases of STEC O26:H11 or O103:H2 infections nationwide from 18 January to 5 April 2022. Fifty cases presented with HUS and two died. Data from supermarket loyalty cards identified frequent purchase of Brand A Type B frozen pizzas. A case–control study confirmed a strong association between the consumption of Brand A pizzas and illness (OR = 116.0; 95% confidence interval (CI): 26.8–501.9). Manufacturing of Brand A Type B pizzas did not include pre-baking of the dough. Isolates from pizza dough and flour samples were indistinguishable from the clinical outbreak strains. On 18 March, the manufacturer recalled the Type B pizzas. While flour is a known STEC vehicle, this outbreak is highly unusual, as cooking of frozen pizzas should eliminate STEC. Further research aiming to understand the origins and persistence of contamination should contribute to improving food safety practices.

**Key public health message**

**What did you want to address in this study and why?**

Shiga toxin-producing *Escherichia coli* (STEC) can cause a severe disease, especially in children and older adults. In 2022, a severe STEC outbreak was identified in France. We investigated the outbreak using several methods, including phone interviews and data from supermarket loyalty cards to identify types of food purchased and consumed, tested food samples and compared food isolates to bacteria identified in patient samples.

**What have we learnt from this study?**

The outbreak had a considerable public health impact: 59 identified cases, 50 of them with a severe form of disease (haemolytic uremic syndrome) and two deaths. We identified a specific line of frozen pizzas of Brand A as the source. These pizzas had a fluffy crust and manufacturing with leavened but not pre-baked dough. Therefore, cooking the pizzas at home may not have reached sufficient temperatures to eliminate the bacteria.

**What are the implications of your findings for public health?**

While risk of STEC contamination in flour is documented in the literature, pizza dough was an unusual outbreak source as typical cooking times and temperatures should eliminate *Escherichia coli*. Our results led to changes in official food control programmes and prevention messages and raise important questions regarding current knowledge and management of *Escherichia coli* risk in flour-based preparations sold raw to consumers.

## Background

Shiga toxin-producing *Escherichia coli* (STEC) infection is the most common aetiology for paediatric haemolytic uraemic syndrome (HUS) [1], which is characterised by haemolytic anaemia, thrombocytopenia and acute renal failure. Young children and older adults are at particular risk for STEC-HUS [2]. In France, STEC surveillance relies on voluntary surveillance of HUS in children and adolescents under 15 years, coordinated since 1996 by Santé publique France (the French Public Health Agency), and voluntary microbiological surveillance coordinated by the National Reference Centre (NRC) for *E. coli* (Institut Pasteur, Paris, France) [2]. Physicians notify suspected paediatric patients with STEC-HUS based on clinical criteria with or without initial detection of Shiga toxin by PCR. Patient samples are sent to the associated NRC (aNRC, Robert Debré Hospital, Assistance Publique – Hôpitaux de Paris (AP-HP), Paris, France) for confirmation, isolation of the bacteria and isolate characterisation by PCR. Thereafter, all isolates are submitted to the NRC for whole genome sequencing (WGS). Microbiological surveillance covers patients other than paediatric HUS, such as those presenting severe clinical symptoms (bloody diarrhoea) or who are at risk of severe disease (young children, older adults, persons with weakened immune systems). Food-borne outbreaks have been mandatorily notifiable since 1987 [3]. Thus, STEC infections may also be notified to regional health authorities if patients are part of a collective food-borne illness event [3]. Over the past 10 years, the annual incidence of paediatric STEC-HUS in France has ranged from 0.8 to 2.2 cases per 100,000 persons under 15 years (87–252 cases notified annually) [1]. Incidence in children under 3 years is highest: 2–5 cases per 100,000 population. Since 2016, the predominant serogroup identified in paediatric HUS cases is O26, accounting for almost half of the cases [1,4,5]. More than 90% of notified paediatric HUS cases are considered sporadic [2]. In France over the past 25 years, most (14/29) documented STEC outbreaks were linked to consumption of undercooked ground beef and raw milk cheeses [1].

## Outbreak detection

From 26 January to 10 February 2022, Santé publique France and the aNRC observed an increase in paediatric STEC infections: cases with HUS notified by physicians or patients with a sample sent to the NRC. Eight of these persons resided in the northern half of France, four were aged > 5 years and three had serogroup O26 (sequence data were pending). On 11 February 2022, due to the increased number of cases, the geographical distribution and the unusual proportion of older cases than typically observed in paediatric HUS surveillance, Santé publique France initiated epidemiological investigations without waiting for typing results.

Here we describe the epidemiological, microbiological and traceback investigations carried out by French authorities to identify the source of infection and discuss the implications for public health and STEC risk management.

## Methods

### Case identification and definitions

Suspected cases investigated by Santé publique France were defined as any notified case of paediatric HUS or HUS with a sample sent to the aNRC from 1 January 2022, and any case of STEC O26 *stx2 eae ehxA* infection confirmed by the aNRC regardless of clinical presentation [2]. As the investigations continued, analysis of STEC isolates first identified a common profile by rapid multilocus variable number tandem repeat analysis (MLVA), confirmed by WGS as the outbreak strain (see Microbiological investigations). At a later stage, patients with STEC O103 infections characterised by a common MLVA and WGS profile were included in the outbreak case definition. Final case definitions included individuals with STEC infection or symptom (diarrhoea or HUS) onset between 1 January and 5 April 2022, and (i) for confirmed cases, isolation of the STEC O26:H11 or STEC O103:H2 outbreak strain, and (ii) for probable cases, development of HUS and an epidemiological link to a confirmed case, but without an isolate (Box).

Box **Final case definitions of Shiga toxin-producing *Escherichia coli* (STEC) infections in a national outbreak associated with frozen pizzas, France, 1 January–5 April 2022**

**Probable case:**

• Development of HUS

AND

• Epidemiological link to a confirmed case

**Confirmed case:**

• Asymptomatic STEC infection OR diarrhoea and/or HUS

AND

• Detection of outbreak strain from a clinical sample

o O26:H11 ST21 with *stx2a*, *eaeβ* and *ehxA* genes belonging to the cgMLST HC5_190514^a^

OR

o O103:H2 ST17, with *stx1a*, *eaeε* and *ehxA* genes belonging to the cgMLST HC5_194142^a^.

HUS: haemolytic uraemic syndrome; ST: sequence type; cgMLST: core genome multilocus sequence typing; HC: hierarchical clustering.^a^ According to the EnteroBase Hierarchical clustering scheme (https://enterobase.readthedocs.io/en/latest/features/clustering.html)

### Epidemiological investigations and hypothesis generation

Santé publique France epidemiologists contacted the families of the patients by phone to conduct interviews using a trawling STEC questionnaire with questions on patient demographics, clinical characteristics, environmental and food exposure data during the 7 days before symptom onset (15 days before HUS diagnosis for patients presenting without diarrhoea). Food exposures included consumption of meat, dairy products, raw fruits and vegetables, flour-based preparations containing undercooked or uncooked flour and beverages (juices and water). For each food item consumed, we collected details on brands, places and dates of purchases. Families also provided supermarket loyalty card numbers for traceback investigations. Initial interviews and supermarket loyalty card data identified several food items with reported consumption or purchase by most patients but failed to identify a common source that could explain the outbreak. Therefore, we conducted a second round of interviews to specify additional food exposures identified in particular from supermarket loyalty card purchases.

### Case–control study

We conducted a case–control study to test hypotheses generated from case interviews. Controls were anonymously recruited through a cohort of adults registered on Grippenet/Covidnet who participate in online population-based surveillance for influenza-like illness. Grippenet/Covidnet participants with children and adolescents aged 2–15 years were invited by email to complete an anonymous online questionnaire exploring food consumptions of one child living in their household during the prior 7 days. If there was more than one child in the household, the questionnaire was completed for the child with the next birthday. Age, sex and region were compared between case patients and controls using t test and Pearson’s chi square test. Food consumption frequencies were compared between cases and controls using Pearson’s chi square test. We used univariable logistic regression models to calculate odds ratios (OR) with 95% confidence intervals (CI) as measures of association between each variable and illness. We then used multivariable logistic regression models to adjust associations for age (0–5, 6–10 and 11–17 years), sex and region (13 regions). Collinearity between food exposures was assessed using Cramer’s V correlation coefficient (range: 0–1). Collinearity was deemed unacceptable when variables had strong (0.40 ≤ V < 0.80) or very strong (0.80 ≤ V < 1) correlation [6]. Data were analysed using Stata version 16 (StataCorp, College Station, the United States (US)).

### Product traceback investigation

Food traceback investigations, including obtaining supermarket loyalty card data, were conducted by the General Directorate for Food (DGAL) and the General Directorate for Competition Policy, Consumer Affairs and Fraud Control (DGCCRF). After having analysed case interview and supermarket loyalty card data, we identified several frequently consumed or purchased at-risk food items. In the traceback investigations, we included batches of the identified products by contacting restaurants, retailers and their suppliers. In the absence of a common identified product, the manufacturers were asked to identify the raw materials used in the identified batches. The production conditions and food safety controls of the relevant establishments were also verified. Samples from suspected food vehicles available in cases’ homes and in manufacturing plants were collected.

### Microbiological investigations

Stool samples were sent to the aNRC where initial STEC screening by PCR (*stx1, stx2, eae*) and isolation by culture were performed [2]. All isolates were characterised by PCR (10 major serogroups, *stx1, stx2, eae, ehxA*) [7]. Food samples obtained in traceback investigations were analysed for STEC according to an adapted CEN ISO/TS 13 136: 2012 at the National Reference Laboratory for *E. coli*. More precisely, 25 g of a food sample was enriched in buffered peptone water broth. Thereafter, serogroup markers and virulence genes (*stx1*, *stx2*, *eae, ehxA*) were detected by PCR from enrichment broths. For suspect samples, the presence of STEC was confirmed by isolation from selective agar plates. Virulence genes and serotypes were then characterised by PCR. Both clinical and food isolates underwent MLVA analysis at the aNRC for a more rapid isolate comparison, as WGS results are obtained ca 10 days after isolation [8]. All isolates were sequenced at the NRC at the *Plateforme de microbiologie mutualisée* (P2M) from the Pasteur International Bioresources network (PIBnet, Institut Pasteur, Paris, France). The MagNAPure 96 system (Roche Diagnostics, Basel, Switzerland) was used for DNA extraction, libraries were prepared using the Nextera XT kit (Illumina, San Diego, US) and sequencing done with the NextSeq 500 system (Illumina) [9]. The determination of serotype (O and H antigens), virulence genes (*stx, eae, ehxA, saa, aggR* and *subA*), acquired antimicrobial resistance genes and multilocus sequence typing (MLST) were performed using tools available at the Centre for Genomic Epidemiology (https://genomicepidemiology.org/services/) [10,11]. Phylogenetic analysis was performed by core genome (cg) MLST hierarchical clustering (cgMLST HC) and cg single nucleotide polymorphism (SNP)-based maximum-likelihood phylogenetic trees (with the ‘Create SNP Project’ tool), integrated into EnteroBase (https://enterobase.warwick.ac.uk/) [10]. The NRC informed Santé publique France of the results.

## Results

### Outbreak case description

**Figure 1:**
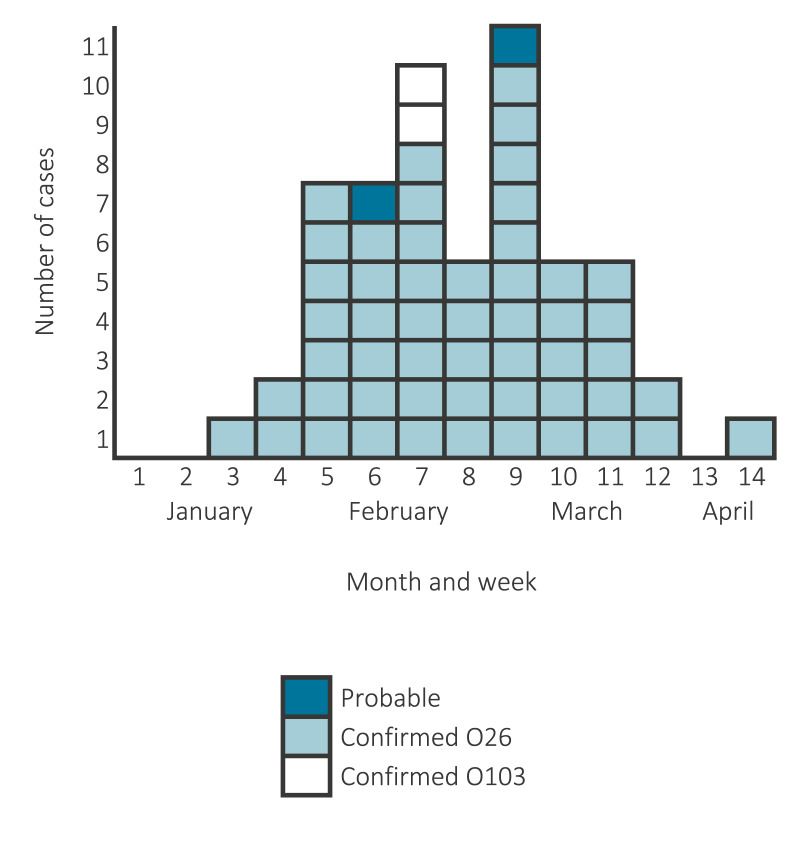
Number of confirmed and probable cases in an outbreak associated with Shiga toxin-producing *Escherichia coli* O26:H11 and O103:H2 infection, by week of illness onset, France, January–April 2022 (n = 56)^a^

^a^ Three asymptomatic confirmed cases were not included in the outbreak curve.

We identified 57 confirmed cases (55 STEC O26 and two STEC O103) and two probable cases. Symptom onset spanned from 18 January (week 3/2022) to 5 April (week 14/2022) (Figure 1), with the first peak in week 7 and the second peak in week 9. The cases were nationwide but predominantly resided in the northern half of France (44/59). The median age of the cases was 6 years (interquartile range (IQR): 3–10; range: 9 months–17 years), and 33 of 59 were males. Only one confirmed case (asymptomatic infection) was an adult, a parent in a household with a paediatric case.

Fifty paediatric cases developed HUS (47 preceded by diarrhoea) and at least 10 of them presented severe neurological involvement. Two of the cases with neurological involvement died. Six cases without HUS presented with diarrhoea, including one with severe colitis. Three cases were asymptomatic. Overall, 18 cases were clustered within eight households. Nine other cases reported diarrhoea in their household members without confirmed aetiology.

### Hypothesis generation

In week 9, via trawling STEC questionnaires we identified consumption of ground beef burgers for 30 of 35 cases, and consumption of food from fast food chain W for 23 of 35 cases. However, traceback investigations failed to identify a common origin for these food items. Ground beef burgers consumed by cases were from different manufacturers and preparations (frozen, fresh), and no common source of raw materials was identified. In parallel, we continuously analysed data from supermarket loyalty cards. This included identifying common food types purchased by more than half of cases and when possible, common product brands. Through this analysis, we identified purchase of pizzas for 21 of 28 cases with an available card, of which 17 were Brand A Type B frozen pizzas with various toppings. At the time when Brand A Type B frozen pizzas were identified, no common brand was identified for the other frequently purchased food items such as ground beef burgers, cow’s or goat’s milk cheese. A second round of interviews of confirmed cases from 4 March (week 9) confirmed consumption of Brand A pizzas for 15 of 22 cases. In parallel, we developed a case–control study to test the hypothesis that consumption of Brand A pizza was associated with illness, compared with the other frequently consumed or purchased items, including ground beef burgers, ground beef, certain raw cow’s and goat’s milk cheeses, salads and fast food.

### Case–control study

In the case–control study, we included the 33 symptomatic confirmed cases identified at the time of the case–control study (week 9) and 514 controls. Mean age of case patients was 5 years (IQR: 3–10) and 9 years (IQR: 6–13) for controls (p < 0.001). Seventeen of the 33 case patients and 285 (56%) of 512 controls were males (p = 0.841). Geographical distribution differed between case patients and controls (p < 0.001) with case patients concentrated in four of 13 regions and controls distributed nationwide.

In univariable analysis, illness was associated with consumption of ground beef burgers (OR = 3.2; 95% CI: 1.4–7.6), ground beef of no particular brand (OR = 9.4; 95% CI: 4.1–21.8), pizza of no particular brand (OR = 6.3; 95% CI: 2.6–15.1), Brand A pizza (OR = 57.3; 95% CI: 19.6–167.9) and eating at a fast food restaurant (any chain) (OR = 7.6; 95% CI: 3.5–16.5) (Table). The strong collinearity between consuming Brand A pizza and ground beef among cases (Cramer’s V = 40.4%) precluded a multivariable analysis testing both exposures. Associations remained significant for all food exposures after adjusting for age, sex and region of residence (Table). There was no association with a particular brand of ground beef or fast food chain. In contrast, case patients had a 116-fold (95% CI: 26.8–501.9) higher odds to have eaten Brand A pizza than controls.

**Table:** Selected exposures among confirmed cases and controls in an investigation of an outbreak with Shiga toxin-producing *Escherichia coli* O26:H11 and O103:H2 infection, France, January–April 2022^a^

Exposure	Cases (n = 33)			Controls (n = 514)				OR	95% CI	aOR^b^	95% CI
	Exposed (n)	Respondents (n)	%	Exposed (n)		Respondents (n)	%				
Ground beef											
Ground beef burger	25	32	78	257		489	53	3.2	1.4–7.6	3.8	1.5–9.6
Ground beef	22	30	73	114		505	23	9.4	4.1–21.8	14.8	5.4–40.6
No ground beef	11	30	37	397		489	81	Reference			
Brand X	8	30	27	10		489	2	28.9	9.6–87.3	30.0	7.4–121.9
Brand Y	5	30	17	8		489	2	22.6	6.4–80.2	25.3	4.7–135.5
Other brand	6	30	20	74		489	15	2.9	1.1–8.2	4.5	1.4–14.1
Dairy products											
Goat cheese	11	33	33	156		497	31	1.1	0.52–2.3	1.3	0.54–2.9
Raw cow’s milk cheese	9	31	29	75		507	15	2.4	1.04–5.3	2.2	0.90–5.5
Pizza											
Pizza	21	28	75	164		508	32	6.3	2.6–15.1	7.7	2.9–19.9
No pizza	7	27	26	344		441	78	Reference			
Brand A	14	27	52	12		441	3	57.3	19.6–167.9	116.0	26.8–501.9
Other brand	6	27	22	85		441	19	4.1	1.4–11.9	4.5	1.4–14.7
Fast food											
Any fast food chain	23	33	70	118		509	23	7.6	3.5–16.5	9.1	3.9–21.6
No fast food	10	33	30	391		509	77	Reference			
Chain W	17	33	52	75		509	15	8.9	3.9–20.1	10.4	4.1–26.4
Other brand	6	33	18	43		509	8	5.5	1.9–15.7	6.7	2.0–22.5
Other food											
Raw dough or flour	10	32	31		176	505	35	0.85	0.39–1.8	1.3	0.57–3.1
Salad	10	32	31		277	495	56	0.36	0.17–0.77	0.58	0.25–1.4
Sweet or savoury crepes	10	29	34		286	487	59	2.7	1.2–5.9	3.1	1.3–7.7

aOR: adjusted odds ratio; CI: confidence interval; OR: odds ratio.

^a^ Cases with symptom onset between 18 January and 10 March were included.

^b^ Adjusted for age (age groups: 0–5, 6–10 and 11–17 years), sex and region.

Ultimately, 49 of 59 confirmed and probable cases reported consumption of Brand A pizzas. Supermarket loyalty card data or receipts documented purchase of pizza (any brand) for 46 of 57 confirmed and probable cases with available data, of which 44 of 46 purchased Brand A Type B pizza.

### Product traceback investigation

In week 10, traceback investigations of Brand A Type B pizzas identified a single factory in France that manufactured Type B pizzas in a dedicated production line. The recipes showed that the toppings used on the Brand A Type B pizzas were either cooked or pasteurised. However, Type B was the only Brand A pizza manufactured with no prebaking of the dough but included a leavening step at 35°C, a favourable temperature for STEC growth [12].

In week 11, two unopened Brand A Type B pizzas available at case homes and purchased at the same time as the pizzas consumed before illness were sampled and tested positive for STEC O26 and O103. Pizzas sampled from the manufacturer and flour used for manufacturing the pizzas also tested positive for STEC O26 (pizza dough and flour) and STEC O103 (pizza dough). Several other factories used flour from the same mill, and samples were taken of products including raw cookie doughs, precooked dry flour used by bakeries for handling dough (dusting) and breaded products. STEC was not detected in any of these sampled products.

Brand A did not export Type B pizzas to other countries. Limited distribution outside France by supermarket chains occurred in Europe and French-speaking African countries.

### Microbiological investigations

**Figure 2:**
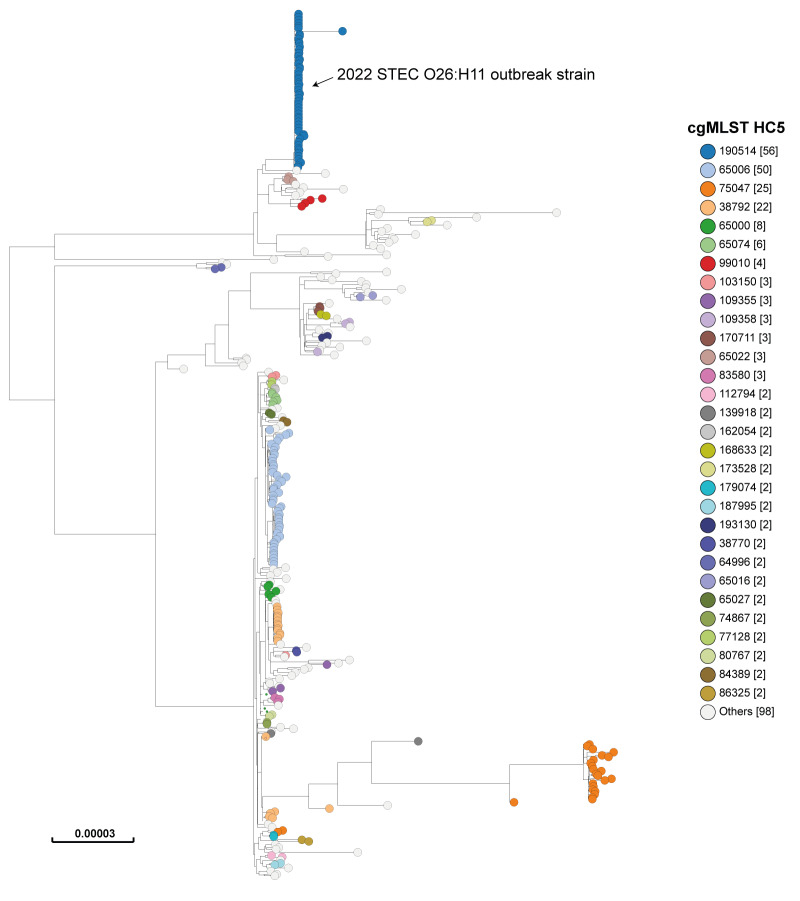
Maximum-likelihood tree of Shiga toxin-producing *Escherichia coli* O26:H11 genomic sequences, France, 2015–2022 (n = 321)

The outbreak strains were *E. coli* serotype O26:H11, ST21, harbouring *stx2a, eaeβ* and *ehxA* virulence genes, and O103:H2, ST17, harbouring *stx1a*, *eaeε*, and *ehxA.* The O26 outbreak strain displayed a unique type “190514”, and the O103 a unique type “192142” by hierarchical clustering of cgMLST data at ≤ 5 allelic differences (HC5). A phylogenetic analysis relying on core genome SNPs confirmed the similarity of each of the outbreak strains. Figure 2 illustrates the maximum-likelihood tree for O26:H11, with the outbreak cluster HC5_190514 (Figure 2). The maximum-likelihood tree for O103:H2 illustrating the outbreak cluster HC5_192142 is provided in Supplementary Figure S1. Also, WGS confirmed clustering between O26 isolates from pizza dough and patients (Figure 2) and between the O103 isolate from pizza dough and the O103 isolates from the two patients, as presented in Supplementary Figure S1. Supplementary Table S1 presents genomic characteristics and the European Bioinformatics Institute European Nucleotide Archive (EBI-ENA; https://www.ebi.ac.uk/ena/browser/home) accession numbers for each genome.

cgMLST: core genome multilocus sequence typing; HC: hierarchical clustering; ST: sequence type; STEC: Shiga toxin-producing *Escherichia coli*.

Phylogeny based on 5,911 SNPs from 321 STEC O26:H11, ST21, HC50_574 genomes, exclusively from the National Reference Centre for *Escherichia coli* collection (320 human source genomes and 1 food source genome isolated from pizza dough) as of 5 May 2022. Reference genome used for mapping: 202201037.

## Outbreak control measures

As the number of confirmed and suspected cases increased and the vehicle of transmission and source of the outbreak were not yet identified, public health authorities issued press releases in February and March 2022 informing the public of the outbreak and communicating recommendations related to general STEC prevention. Recommendations included, but were not limited to, washing hands before meals, cooking ground beef thoroughly, avoiding consumption of unpasteurised dairy products, in particular for children under 5 years, avoiding consumption of raw or undercooked flour-based preparations and cooking them thoroughly (e.g. pizza, cookie dough, cake batter) [13].

Following isolation of STEC O26 and O103 in Brand A Type B pizzas, Brand A issued a voluntary recall and withdrawal from the market on 18 March 2022 (week 11) of all Type B pizzas manufactured since 1 June 2021 [14]. More than 10,000 controls on the effectiveness of the withdrawal in stores were carried out by the DGAL, the DGCCRF and their regional authorities. The Brand A factory that produced Type B pizzas was subsequently closed.

Public health authorities led an intense media campaign in French newspapers, radio and TV to ensure that information on the recall reached as many people as possible, as there was a high risk that the frozen pizzas could remain in consumers’ freezers and continue to constitute a risk of infection. The number of new suspected cases decreased considerably after the recall (18 March, week 11/2022) (Figure 1). Two confirmed cases reported consumption of Brand A Type B pizzas after the recall, but interviews confirmed purchase before the control measures.

We informed other countries of the outbreak through EpiPulse (2022-FWD-00017; https://www.ecdc.europa.eu/en/publications-data/epipulse-european-surveillance-portal-infectious-diseases), the Early Warning and Response System (https://ewrs.ecdc.europa.eu/) and International Food Safety Authorities Network (InfoSan; https://www.fao.org/food-safety/emergencies/infosan/en), but no potentially related cases were identified.

## Discussion

This was the largest documented STEC-HUS outbreak in France. Evidence from epidemiological, traceback and microbiological investigations confirmed Brand A Type B pizza as the source of the outbreak: (i) most cases reported consumption of Brand A frozen pizzas; (ii) case patients had greater odds of eating Brand A pizza than controls; (iii) documented purchase of Brand A Type B pizza was found for the majority of cases reporting consumption of Brand A frozen pizzas and (iv) Brand A Type B pizza dough samples and flour used for manufacturing tested positive for STEC outbreak strains. Fifty-nine confirmed and probable cases were identified in this outbreak; 50 of them were children and adolescents with HUS. This likely underestimates the true number of STEC infections of the outbreak. As with any food-borne outbreak, there is a higher probability of identifying severe cases than asymptomatic or mild cases. For STEC in France, this is due in part to the surveillance system focusing on paediatric STEC-HUS cases, but also more generally that medical consultation for gastrointestinal illness depends on factors such as symptom severity and duration and patient demographics, with a greater tendency to consult for children and older adults. While sequencing is routine in the French STEC surveillance, the delay from sample collection to sequencing results is around 3 weeks. As the predominant serogroup of the characterised isolates in this outbreak was O26, the most frequent serogroup in paediatric STEC-HUS in France, we applied MLVA which takes around 24 h, for initial characterisation. This was key in identifying a common profile and restraining case definitions for data analyses to cases more likely to be linked to a common source. Sequencing confirmed that isolates with the common MLVA profile belonged to the same cluster defined by cgMLST and hierarchical clustering, and by SNP analysis.

Although uncooked or undercooked dough has been associated with several STEC-HUS outbreaks [15], pizzas, particularly frozen pizzas, are an unusual vehicle for STEC infection as typical cooking times and temperatures during manufacturing and at home are generally sufficient to eliminate the bacterium [16]. This unexpected food vehicle, combined with frequent reported consumption of several known at-risk STEC food items, complicated the investigations. In France, the use of supermarket loyalty card data in food-borne outbreak investigations has become common [17]. Such data, obtained in a timely matter (generally within 2 days of interviews), were essential for collecting precise data on commonly purchased food items, and in this outbreak ultimately led to the identification of frozen pizzas as a suspected vehicle. Although the trawling STEC questionnaires are extensive and include a section on flour-based preparations containing undercooked or uncooked flour, pizza was not specifically included, nor was it identified by parents as an undercooked flour-based preparation. After identification of a specific brand and type of frozen pizzas as a suspected vehicle through supermarket loyalty card purchases, it was possible to conduct the necessary complementary investigations to confirm this hypothesis: secondary interviews provided strong epidemiological evidence for pizzas as the vehicle of contamination, with 49 of 59 cases ultimately reporting consumption of the Brand A frozen pizzas. The case–control study and the traceback and food sampling data reinforced the plausibility of this hypothesis, highlighting the importance of complementary data from all aspects of investigations to identify, test and confirm hypotheses, particularly when faced with an unusual food vehicle.

The case–control study was key in testing the different hypotheses generated from the trawling questionnaires and could be initiated rapidly by using a web-based population cohort, an approach already used for a previous food-borne outbreak investigation [18].

Microbiological analyses of pizza dough and flour confirmed STEC contamination with the outbreak strains. Flour has been described as a vehicle for potentially pathogenic STEC strains, and flour and flour-based products have been previously linked to several STEC outbreaks, in particular in North America, although to our knowledge, frozen pizza dough has not previously been implicated [19-23]. In the US, flour generally includes wheat from different sources, making identification of the source of the contamination difficult [15]. Following this outbreak in France, the authorities investigated the complete production chain of pizza dough, including flour harvesting, storage, processing and dough manufacturing, to identify potential sources of STEC contamination and persistence.

Brand A Type B pizza, which did not have prebaked dough, was advertised as having an extra fluffy crust on its packaging. This characteristic was accompanied by cooking instructions at higher temperatures and longer duration (15–19 min at 240°C in a preheated traditional oven or 16–18 min at 230°C in a preheated convection oven) compared with the instructions indicated on the packaging for other brands of frozen pizzas on the market that contain dough pre-baked at manufacturing (11–14 min at 200–220°C). These temperatures might not be reached and/or maintained by all ovens commonly used at home. Additionally, the dough and toppings become visually appetising before reaching the indicated time and temperature ranges. Together, these factors might have contributed to the consumption of dough with certain parts that had not reached sufficient temperatures to eliminate STEC risk, particularly due to the thicker, fluffy crust. However, the persistence of STEC in these cooking conditions remains unexplained as typically cooking to > 70°C is sufficient to eliminate STEC risk. Attempts to describe in more detail the potential for undercooking through the case interviews yielded limited and inconclusive data, as families had difficulties identifying if the product was well cooked. The interviews did not identify consumption of the raw dough.

## Conclusion

This outbreak is important for public health, food safety authorities and recommendations regarding food safety practices, such as Hazard Analysis Critical Control point (HACCP), as it demonstrates the extent of STEC risk in flour and flour-based foods. The DGAL integrated sampling of flour and flour-based preparations into their annual food surveillance programme, and Santé publique France revised STEC prevention messages to explicitly include sufficient cooking of flour-based products with examples including, but not limited to, pizza dough. Completing a risk analysis by food safety agencies is a next important step to aid in adapting and developing recommendations regarding food safety practices during manufacturing of these products and to tailor consumer recommendations.

## Data Availability

Aggregated data presented in the manuscript or supplementary material are available from the corresponding author upon reasonable request. Sequence data accension numbers are available in Supplementary Table S1 and SNP trees are available on EnteroBase at the following link: https://enterobase.warwick.ac.uk/species/ecoli/snp_project/86660

## Supplementary Data

317000 Supplementary Material
