# Supplementary material for: Nationwide outbreak of Shiga toxin-producing Escherichia coli infections associated with frozen pizzas, France, 2022
Source: Euro Surveill. 2026 Feb 26;31(8):2500506. doi: 10.2807/1560-7917.ES.2026.31.8.2500506 (PMC13074260; doi:10.2807/1560-7917.ES.2026.31.8.2500506)
Supplement: Supplementary Material [file 25-00506_JONES_Supplement.pdf]

Supplementary Figure 1 : Maximum-likelihood tree of Shiga toxin-producing *Escherichia coli* O103:H2 genomic sequences, 2001–2022 (n = 200)

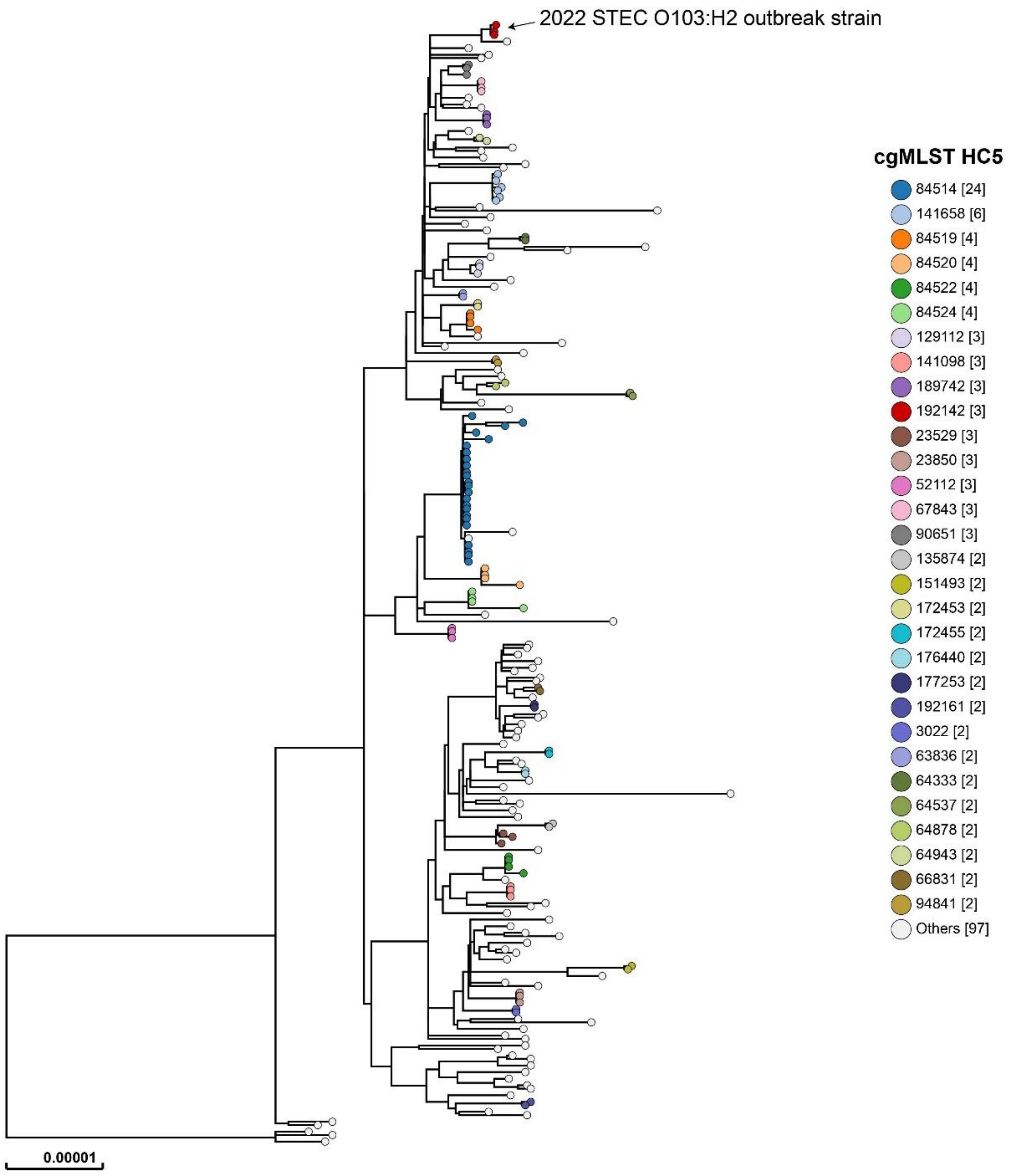

cgMLST: core genome multilocus sequence typing; HC: hierarchical clustering; ST: sequence type; STEC: Shiga toxin-producing *Escherichia coli*.

Phylogeny based on 4,193 SNPs from 200 STEC O103:H2, ST17, HC50_1985 genomes, available on EnteroBase as of April 22, 2022. It included 16 genomes from the National reference centre for *E. coli* collection (15 human source genomes and 1 food source genome). Reference genome used for mapping: 20220119

Supplementary Table 1 : Genomic characteristics and the European Bioinformatics Institute European Nucleotide Archive (EBI-ENA) accession numbers for each genome of isolates from an outbreak with Shiga toxin–producing *Escherichia coli* O26:H11 and O103:H2 linked to consumption of frozen pizzas, France, January–April 2022

This supplementary material is hosted by Eurosurveillance as supporting information alongside the article [Nationwide outbreak of Shiga Toxin-Producing *Escherichia coli* infections associated with frozen pizzas – France, 2022], on behalf of the authors, who remain responsible for the accuracy and appropriateness of the content. The same standards for ethics, copyright, attributions and permissions as for the article apply. Supplements are not edited by Eurosurveillance and the journal is not responsible for the maintenance of any links or email addresses provided therein.

**Isolate**	**Serotype**	**MLST**	**stx1**	**stx2**	**eae**	**ehxA**	**ehxA/hlyA**	**ENA accession nos.**
202200751	O26:H11	ST21	-	stx2a	beta	ehxA	+	ERR14097261
202200858	O26:H11	ST21	-	stx2a	beta	ehxA	+	ERR14097263
202200865	O26:H11	ST21	-	stx2a	beta	ehxA	+	ERR8778625
202200866	O26:H11	ST21	-	stx2a	beta	ehxA	+	ERR14097267
202200867	O26:H11	ST21	-	stx2a	beta	ehxA	+	ERR14097268
202201037	O26:H11	ST21	-	stx2a	beta	ehxA	+	ERR14097272
202201038	O26:H11	ST21	-	stx2a	beta	ehxA	+	ERR14097273
202201040	O26:H11	ST21	-	stx2a	beta	ehxA	+	ERR14097274
202201042	O26:H11	ST21	-	stx2a	beta	ehxA	+	ERR14097275
202201043	O26:H11	ST21	-	stx2a	beta	ehxA	+	ERR14097276
202201045	O26:H11	ST21	-	stx2a	beta	ehxA	+	ERR14097277
202201046	O26:H11	ST21	-	stx2a	beta	ehxA	+	ERR14097278
202201047	O26:H11	ST21	-	stx2a	beta	ehxA	+	ERR14097279
202201195	O26:H11	ST21	-	stx2a	beta	ehxA	+	ERR14097280
202201196	O26:H11	ST21	-	stx2a	beta	ehxA	+	ERR14097281
202201198	O26:H11	ST21	-	stx2a	beta	ehxA	+	ERR14097282
202201199	O26:H11	ST21	-	stx2a	beta	ehxA	+	ERR14097283
202201200	O26:H11	ST21	-	stx2a	beta	ehxA	+	ERR14097284
202201201	O26:H11	ST21	-	stx2a	beta	ehxA	+	ERR15682474
202201202	O26:H11	ST21	-	stx2a	beta	ehxA	+	ERR14097285
202201204	O26:H11	ST21	-	stx2a	beta	ehxA	+	ERR14097286
202201387	O26:H11	ST21	-	stx2a	beta	ehxA	+	ERR14097287
202201388	O26:H11	ST21	-	stx2a	beta	ehxA	+	ERR14097288
202201392	O26:H11	ST21	-	stx2a	beta	ehxA	+	ERR14097289
202201393	O26:H11	ST21	-	stx2a	beta	ehxA	+	ERR14097290
202201394	O26:H11	ST21	-	stx2a	beta	ehxA	+	ERR14097291
202201395	O26:H11	ST21	-	stx2a	beta	ehxA	+	ERR14097292
202201397	O26:H11	ST21	-	stx2a	beta	ehxA	+	ERR14097294
202201592	O26:H11	ST21	-	stx2a	beta	ehxA	+	ERR14097295
202201593	O26:H11	ST21	-	stx2a	beta	ehxA	+	ERR14097296
202201594	O26:H11	ST21	-	stx2a	beta	ehxA	+	ERR14097297
202201595	O26:H11	ST21	-	stx2a	beta	ehxA	+	ERR14097298

202201599	O26:H11	ST21	-	stx2a	beta	ehxA	+	ERR14097301
202201600	O26:H11	ST21	-	stx2a	beta	ehxA	+	ERR14097302
202201601	O26:H11	ST21	-	stx2a	beta	ehxA	+	ERR14097303
202201674	O26:H11	ST21	-	stx2a	beta	ehxA	+	ERR14097306
202201675	O26:H11	ST21	-	stx2a	beta	ehxA	+	ERR14097307
202201677	O26:H11	ST21	-	stx2a	beta	ehxA	+	ERR14097308
202201678	O26:H11	ST21	-	stx2a	beta	ehxA	+	ERR14097309
202201679	O26:H11	ST21	-	stx2a	beta	ehxA	+	ERR14097310
202201682	O26:H11	ST21	-	stx2a	beta	ehxA	+	ERR14097312
202201884	O26:H11	ST21	-	stx2a	beta	ehxA	+	ERR14097315
202201887	O26:H11	ST21	-	stx2a	beta	ehxA	+	ERR14097316
202201888	O26:H11	ST21	-	stx2a	beta	ehxA	+	ERR14097317
202201890	O26:H11	ST21	-	stx2a	beta	ehxA	+	ERR14097319
202201891	O26:H11	ST21	-	stx2a	beta	ehxA	+	ERR14097320
202201892	O26:H11	ST21	-	stx2a	beta	ehxA	+	ERR14097321
202202037	O26:H11	ST21	-	stx2a	beta	ehxA	+	ERR14097324
202202038	O26:H11	ST21	-	stx2a	beta	ehxA	+	ERR14097325
202202040	O26:H11	ST21	-	stx2a	beta	ehxA	+	ERR14097326
202202380	O26:H11	ST21	-	stx2a	beta	ehxA	+	ERR14097327
202202386	O26:H11	ST21	-	stx2a	beta	ehxA	+	ERR14097328
202202650	O26:H11	ST21	-	stx2a	beta	ehxA	+	ERR14097330
202212227	O26:H11	ST21	-	stx2a	beta	ehxA	+	ERR14097417
202212228	O26:H11	ST21	-	stx2a	beta	ehxA	+	ERR14097418
202201197	O103:H2	ST17	stx1a	-	epsilon	ehxA	+	ERR9514709
202201590	O103:H2	ST17	stx1a	-	epsilon	ehxA	+	ERR14096096
